# Selection of suitable reference genes for accurate normalization of gene expression profile studies in non-small cell lung cancer

**DOI:** 10.1186/1471-2407-6-200

**Published:** 2006-07-26

**Authors:** Saviozzi Silvia, Cordero Francesca, Lo Iacono Marco, Novello Silvia, Scagliotti Giorgio V, Calogero A Raffaele

**Affiliations:** 1 Department of Clinical and Biological Sciences, University of Torino, Regione Gonzole 10, 10043 Orbassano (Torino), Italy; 2 Department of Informatics, University of Torino, Via Pessinetto 12, 10100 Torino, Italy; 3 Thoracic Oncology Unit, S. Luigi Hospital, Regione Gonzole 10, 10043 Orbassano (Torino), Italy

## Abstract

**Background:**

In real-time RT quantitative PCR (qPCR) the accuracy of normalized data is highly dependent on the reliability of the reference genes (RGs). Failure to use an appropriate control gene for normalization of qPCR data may result in biased gene expression profiles, as well as low precision, so that only gross changes in expression level are declared statistically significant or patterns of expression are erroneously characterized. Therefore, it is essential to determine whether potential RGs are appropriate for specific experimental purposes. Aim of this study was to identify and validate RGs for use in the differentiation of normal and tumor lung expression profiles.

**Methods:**

A meta-analysis of lung cancer transcription profiles generated with the GeneChip technology was used to identify five putative RGs. Their consistency and that of seven commonly used RGs was tested by using Taqman probes on 18 paired normal-tumor lung snap-frozen specimens obtained from non-small-cell lung cancer (NSCLC) patients during primary curative resection.

**Results:**

The 12 RGs displayed showed a wide range of Ct values: except for rRNA18S (mean 9.8), the mean values of all the commercial RGs and ESD ranged from 19 to 26, whereas those of the microarray-selected RGs (BTF-3, YAP1, HIST1H2BC, RPL30) exceeded 26. RG expression stability within sample populations and under the experimental conditions (tumour versus normal lung specimens) was evaluated by: (1) descriptive statistic; (2) equivalence test; (3) GeNorm applet. All these approaches indicated that the most stable RGs were POLR2A, rRNA18S, YAP1 and ESD.

**Conclusion:**

These data suggest that POLR2A, rRNA18S, YAP1 and ESD are the most suitable RGs for gene expression profile studies in NSCLC. Furthermore, they highlight the limitations of commercial RGs and indicate that meta-data analysis of genome-wide transcription profiling studies may identify new RGs.

## Background

Lung cancer remains the leading cause of cancer-related death in Europe and North America and accounts for nearly 30% of the total [1,2]. Despite advances in treatment, the prognosis of non-small cell lung cancer (NSCLC) is poor: only 5–15% of patients survive 5 years after diagnosis, mainly in function of the initial stage of the disease [3]. Real-time quantitative PCR (qPCR) is the foremost method of choice for accurate determination of transcripts amounts. It has recently been applied in lung cancer studies to quantify the expression level of predictive and/or prognostic target genes [4,5].

In gene expression studies, qPCR data need to be normalized to remove nonspecific variability associated with differences in RNA quantity and quality, usually by relative quantification whereby the expression level of the target gene is determined relatively to another gene transcript, the so-called reference gene (RG), and the results are expressed as a target to reference ratio [6]. The reliability of normalized data is highly dependent on RG robustness. An ideal RG should be constitutively expressed and characterized by stable expression in different samples/experimental conditions.

Genes considered to be valid RGs in semi-quantitative techniques (eg. Nothern Blot) may be less reliable when highly sensitive qPCR is used [7]. Identification of more sensitive RGs and their validation within specific biological conditions are thus critical issues in qPCR studies [8].

Microarray data can be used to identify potential RGs by modeling the expression data to select those with the most stable expression [9-11]. Most lung cancer qPCR studies use commercial RGs, such as glyceraldehyde-3-phosphate dehydrogenase (GAPDH) [12], β-actin (ACTB) [13], TATA-binding protein [14], β-microglobulin [15], cytochrome oxidase II [16], 18S ribosomal RNA (rRNA18S) [4]. Their reliability in this contex, however, has not been demonstrated. Furthermore, it is increasingly evident that in many experimental situations the use of GAPDH or ACTB is inappropriate due to their high variability [10,17-20]. Thus, the aim of the present study was the selection and validation of new RGs for the differentiation of normal and tumor lung specimens. Five putative RGs (ESD, BTF3, HIST1H2BC, RPL30, YAP1) selected from a meta-analysis of geneChip experiments [21] were validated along with ACTB, GAPDH, RNA18S, PPIA, PGK1, RPLP0 and POLR2A by qPCR assessment of their expression levels on 18 paired normal lung and NSCLC samples. Expression changes between and within these two classes were investigated to define the most stable and comprehensive RGs.

## Methods

### Samples

Primary tumour samples and paired normal lung tissues obtained from 18 NSCLC patients (11M, 7F) aged 41–79 (mean 62) during primary curative resection (not preceded by radiotherapy or chemotherapy) at the San Luigi Hospital, Orbassano (Italy) between December 2003 and March 2004 were immediately snap-frozen in liquid nitrogen and stored at -80°C until RNA extraction. The histological subtypes were: 7 adenocarcinomas (ADCA, 38.9%), 10 squamous cell carcinomas (SQCA, 55.6%) and 1 broncoalveolar carcinoma (BAC, 5.5%). Prior written and informed consent was obtained from each patient. The study was approved by the appropriate ethical review board. The guidelines for good clinical practice and the recommendations of the Declaration of Helsinki for biomedical research involving human subjects were followed.

### RNA extraction and cDNA synthesis

Total RNA (totRNA) was isolated from lung specimens with the RNeasy 96 Kit and Biorobot 8000 (Qiagen, Hilden, Germany) according to the manufacturer's instructions. RNA was extracted from 15–25 mg and 60–80 mg of tumor and normal lung tissues specimens respectively. To take account of the expression variability due to cellular heterogeneity, totRNA was extracted from a biological duplicate of both normal and tumour specimens. Genomic DNA contaminations were removed by on-column-DNAseI treatment. totRNA was then quantified with an Agilent 2100 Bioanalyzer (Agilent Technologies, Palo Alto, CA, USA) and stored at -80°C. Two μgr totRNA were finally retro-transcribed with random hexamer primers and Multiscribe Reverse transcriptase (MMLV) contained in the High Capacity cDNA Archive Kit (Applied Biosystems, Foster City, CA), in accordance with the manufacturer's suggestions.

### Real-time PCR

Expression levels of the 5 putative and 7 commercial RGs (ACTB, *β-actin*; GAPDH, *glyceraldehyde-3-phosphate dehydrogenase*; PGK1, *phosphoglycerate kinase 1*; POLR2A, polymerase *RNA II polypeptide A, 220kDa*; PPIA, *cyclophilin A*; rRNA 18S, *18S ribosomal RNA*; RPLP0, *ribosomal protein, large, P0*; ESD, esterase *D/formylglutathione hydrolase*; BTF3, *basic transcription factor 3*; HIST1H2BC, *histone 1 H2bc*; RPL30, *ribosomal protein L30*; YAP1, Yes-*associated protein 1, 65kDa*) and 3 target genes (BRCA1, *breast cancer 1 early onset*; ERCC2, *excision repair cross-complementing rodent repair deficiency, complementation group 2*; RRM2, *ribonucleotide reductase M2 polypeptide*) were evaluated with TaqMan Probes commercially available as "Assay on Demand" (Applied Biosystems, Foster City, CA, USA) with optimised primer and probe concentrations (Table 1). Quantitative PCR (qPCR) was performed on an ABI PRISM 7900HT Sequence Detection System (Applied Biosystems, Foster City, CA, USA) in 384 well plates assembled by Biorobot 8000 (Qiagen, Hilden, Germany) and the reaction was performed in a final volume of 20 μl. All qPCR mixtures contained 1 μl of cDNA template (corresponding approximately to 20 ng retro-transcribed totRNA), 1× TaqMan Universal PCR Master Mix (2×) (Applied Biosystems, Foster City, CA, USA) and 1× Assay-on-Demand Gene Expression Assay Mix (20×). Cycle conditions were as follows: after an initial 2-min hold at 50°C to allow AmpErase-UNG activity, and 10 minutes at 95°C, the samples were cycled 40 times at 95°C for 15 sec and 60°C for 1 minute. Baseline and threshold for Ct calculation were set manually with the ABI Prism SDS 2.1 software. Automation allowed negligible intra-assay variation (≤5% CV) and low inter-assay variation (≤10% CV) when evaluated on raw linear expression quantities.

### Data analysis

Meta-analysis of expression data was performed by using affy, MergeMaid and genefilter libraries implemented in Bioconductor [22]. Statistical analyses were performed with R [23]. Gene differential expression was considered significant with an α = 0.05 and p-value <0.05. PCA (Principal Component Analysis, [24]) and agglomerative hierarchical clustering were performed with TMEV software [25]. Assays on Demand's PCR efficiencies, evaluated by cDNA dilution curves, ranged between 91% (rRNA18S, RPL30) and 100% (POL2RA, ESD) with an average value of 96%. Since PCR efficiencies were high and comparable we used the theoretical value of 2 (100%) for gene expression quantification. So, fold changes in expression levels between the normal and tumor paired samples were evaluated with 2^-Δ'Ct ^[7]. The expression of each target gene was reported as 2^-ΔΔCt^[6].

The GeNorm Software [26] was obtained from the Gene Quantification Home Page [27].

Biological replicates were treated as independent samples in RGs raw Ct distribution analysis and geNorm analysis. Their average Ct value was used in Δ'Ct calculation (i.e for each sample mean Ct_tumor-mean Ct_normal) applied to evaluate the RGs expression stability with equivalence test. We also averaged the ΔCt value (Ct target-Ct reference) of biological replicates to generate the mean ΔΔCt values used in PCA and agglomerative hierarchical clustering according to the Euclidean distance metric and average linkage method.

## Results

### Arrays meta-analysis

Five putative RGs were selected from a meta-analysis of a subset of lung tissue transcription profilings [21]. To secure a balanced set of tissues, the analysis focused on 17 normal samples, 18 ADCA (randomly selected from a total of 127), 21 SQCA, 20 pulmonary carcinoids and 6 small-cell lung carcinomas. After RMA intensity calculation [28] and quantile normalization [29], an intensity filter was applied to remove all probe sets with intensity ≤100 in all experiments.

Tumour/normal gene expression similarity was measured with the "integrative correlation" (IC) estimation, developed by Parmigiani et al. [30]. This reflects the general consistency of gene expression among the different tissues since it is generated using gene-to-gene correlation obtained by all pairwise correlation for all genes across the tissue groups. A variance/IC filter between each tumor group versus the normal tissues was applied to select probe sets with an inter-sample standard deviation (SD) ≤ 0.5 and IC ≥ 0.2. All the 28 top ranked putative RGs displayed a nearly normal distribution of the residuals representing the expression differences between each tumor sample versus normal samples (data not shown). Within this set we selected five candidates RGs (ESD, BTF3, HIST1H2BC, RPL30, YAP1) involved in different biological processes and for which commercial gene expression assays were available.

### RGs expression ranges in lung tissues

**Figure 1:**
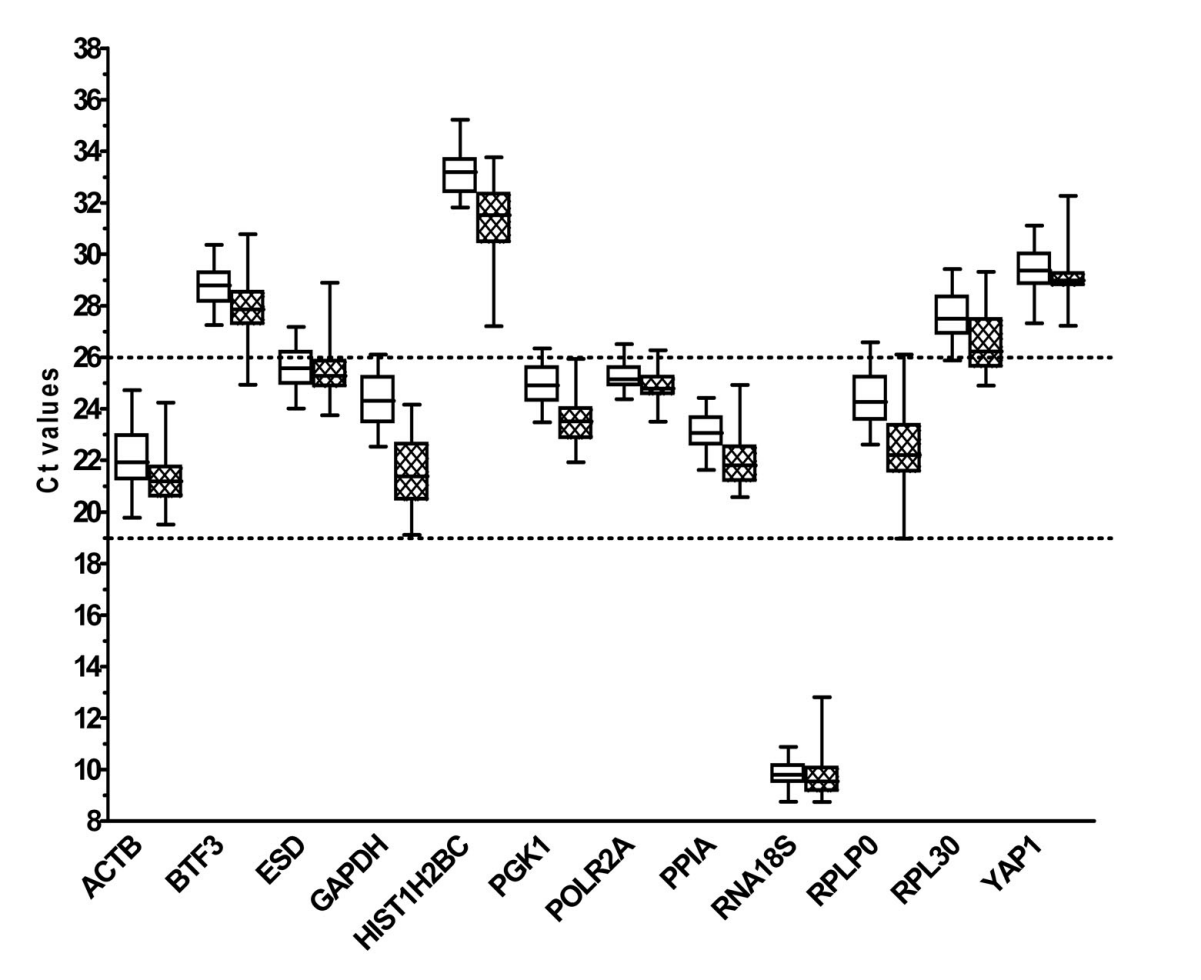
**Expression levels of the 12 candidate RGs in normal and tumor lung tissue samples**. Ct values for each RG are shown as medians (lines), 25th to 75th percentile (boxes) and range (whiskers). Hatched and open boxes represent tumor and normal samples respectively.

The 12 RGs investigated displayed a wide range of Ct values (Figure 1) in separate evaluation of the paired samples. Except for rRNA18S (mean 9.8), the mean values fell into two groups: group A (ESD and the other six commercial RGs): 19–26; group B (the other four microarray-selected RGs): over 26. The microarray-selected RGs (apart from ESD) thus lay in a Ct range not usually covered by commercial RGs and more close to that of our three target genes (i.e. BRCA1, ERCC2, RRM2 with mean Ct values were 31, 29 and 30 respectively). All genes showed a normal distribution pattern proved by Shapiro-Francia fitting procedure (with α = 0.05) both in normal and tumor tissue samples, evaluated with the Bioconductor Nortest package.

### RGs expression stability

To allow accurate quantification a RG should own a stable expression among samples while to warrant a trustworthy quantification it should not be modulated by experimental conditions. Ideally to fulfill both conditions RG raw Ct distribution of both normal and tumor populations should thus be characterized by a narrow range of expression with comparable mean values. With reference to stability of expression in both normal and in tumor samples, the lowest RNA transcription ranges (i.e Ct 75°quantile-Ct 25°quantile) were associated withPOLR2A (normal = 0.73; tumor = 0.69) and rRNA18S (normal = 0.69; tumor = 0.93), whereas the highest ranges were detected for GAPDH (normal = 1.8; tumor = 2.2) and RPLP0 (normal = 1.71; tumor = 1.84). Unfortunately, even when equal amount of RNA input are used, retro-transcription reactions are responsible for most of the variation in the experimental determination of mRNA quantities [31,32]. We are thus unable to estimate how much the technical (i.e. RT efficiency) and the biological (i.e. inter-samples) variability contribute to the measured difference in RNA quantities. Descriptive analysis of raw Ct distributions provides only a rough estimate of RG stability that must be confirmed by other approaches.

To investigate the experimental stability of RG transcription we evaluated the significance of differences in RNA transcription levels between the tumor and normal paired samples, where the latter were used as calibrators in Δ'Ct quantification approach [7,33]. Since in the two-sided t-test the type II errors (false negatives) are uncontrollable, we performed an equivalence test for dependent samples [34] to investigate the null hypothesis of inequivalence H_0_: μ_T_-μ_N _≤ θ_L _and μ_T_-μ_N _≥ θ_U _[35].

**Figure 2:**
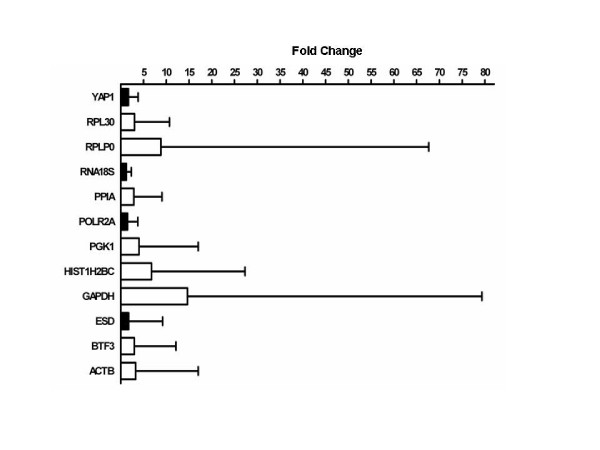
**Fold change in expression levels**. Differences in gene expression levels between tumor and normal sample pairs were represented as average fold change variation (plot) and maximum fold change (bar). Filled columns refer to statistically stable expressed RGs.

The equivalence test showed that only POLR2A, rRNA18S, ESD and YAP1 had a differential expression, between the paired samples, within the equivalence interval [θ_L _= -1; θ_U _= 1], which represents a fold change variation interval [0.5; 2]. All these four genes, unlike the others, were indeed characterized by the lowest average fold change (<1.8) and maximum fold change (<8). Conversely, the greatest variability was associated with GAPDH, RPLP0 and HIST1H2BC (average fold change >6 and maximum fold change ranging from 27 fold for HIST1H2BC to 80 fold for GAPDH) (Figure 2).

**Figure 3:**
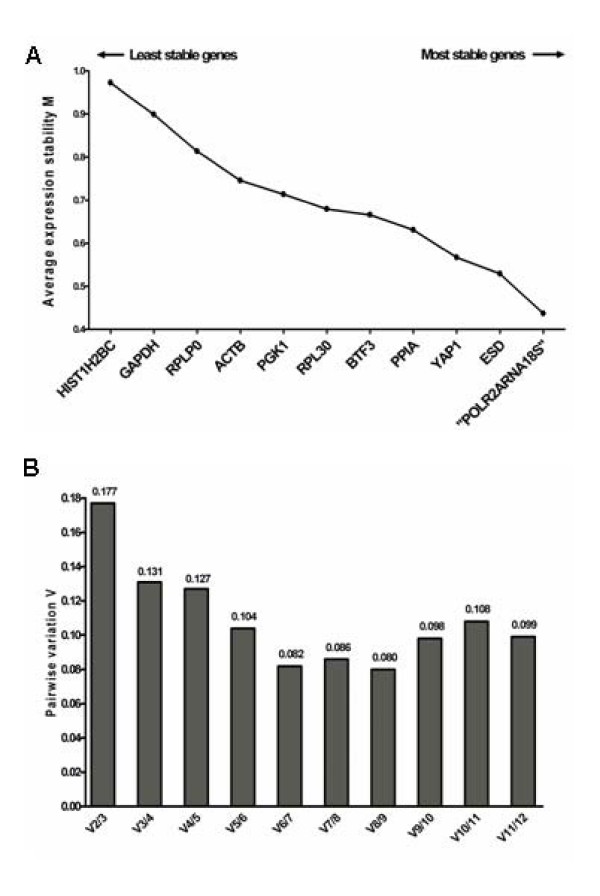
**GeNorm analysis of the 12 candidate RGs**. Selection of the RGs most suitable for normalization in lung cancer gene profiling studies by GeNorm analysis. The results are presented according to the output file of the program. (a) stepwise exclusion of the least stable genes. The x-axis from left to right indicates the ranking of the genes according to their expression stability, while the Y-axis indicates the stability parameter M. (b) determination of the optimal number of RGs for normalization.

Gene expression stability was also investigated with GeNorm software [26], which calculates the measure of gene expression stability (M) of a putative RG based on the average pairwise variation between all investigated RGs. As requested by the software, Ct values were converted to linear expression quantities by 2^-ΔCt ^using the highest expressed sample as calibrator. GeNorm confirmed our results by identifying rRNA18S and POLR2A as the most stable genes (M = 0.436) preceded by ESD and YAP1 (Figure 3a). Results were independent from the histological classification of the lung tumors. The GeNorm program also determines a normalization factor (NF) needed to define the optimal number of RGs required for an accurate normalization strategy. This is calculated from two or more genes with the variable V as the pairwise variation (V_n_/V_n+1_) between two sequential normalization factors (NF_n _and NF_n+1_). The use of at least the three most stable RGs (POL2RA, rRNA18S and ESD) is recommend to improve the accuracy of normalized data, as suggested by a V value below the cut-off of 0.15 (Figure 3b), which was indicated by the authors as the limit beneath which it would not be necessary to include additional reference genes [36].

### Significance of suitable RGs for normalization

**Figure 4:**
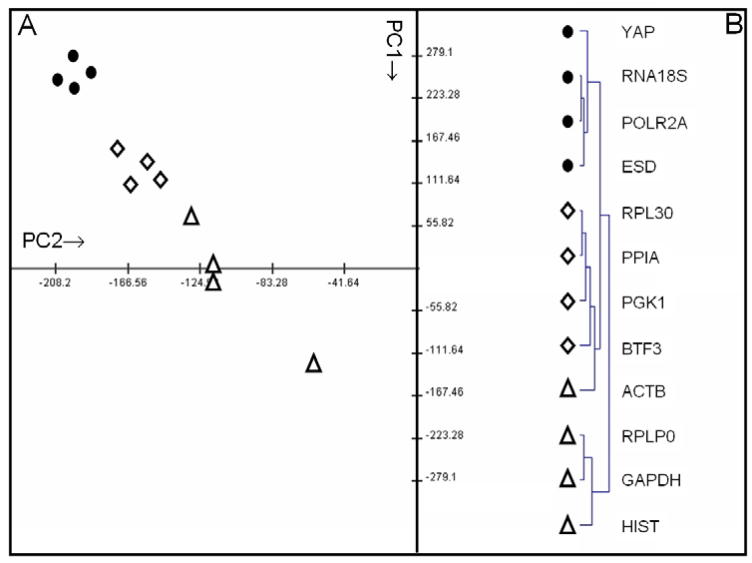
**Targets fold change homogeneity**. PCA and agglomerative hierarchical clustering were used to describe the homogeneity degree of target fold change variation as a function of the reference used. Expression levels of three target genes (RRM2, BRCA1, ERCC2) were normalized with respect to each of the RGs and expressed as -ΔΔCt using normal paired samples as calibrator. A) Small fluctuations in fold change target detection using reliable RGs results in a very limited spread over the PCA space. As expected, POLR2A, rRNA18S, ESD and YAP1 produce the best homogeneous cluster (filled dots). B) Similar results are obtained by hierarchical clustering, where the smallest Euclidean distance is associated with the previously indicated set of genes.

Since normalization of the target genes versus a set of reliable RGs should result in the same fold change variation with minimal fluctuations, Principal Component Analysis [24] and agglomerative hierarchical clustering were used to describe the homogeneity degree of target fold change variation as a function of the reference used (Figure 4). Gene expression levels of three target genes (RRM2, BRCA1, ERCC2) were normalized with respect to each of the available RGs and expressed as -ΔΔCt using normal paired samples as calibrator [6]. Both approaches showed that the most homogeneous group of target fold change evaluations was obtained when the most stable RGs were used for normalization: POLR2A, rRNA18S, YAP1 and ESD (Figure 4A–B filled dots). By contrast, the commonly used RGs ACTB and GAPDH led to inconsistent estimation of fold change (Figure 4A–B open triangles).

## Discussion

This paper describes the first systematic comparison of several conventional and potential RGs and their effectiveness as internal control for relative quantification in lung cancer gene expression profiling. To increase data reliability, we sought to control several variables by (1) using matched pairs of normal and tumor lung samples instead of unpaired samples to minimize inter-individual variations that could arise from genotype profile; (2) analysing biological duplicates of each sample to take into account the expression variability due to cellular heterogeneity in both normal and tumor lung tissues; (3) employing a liquid handling robot for most of the technical procedures (i.e totRNA extraction and qPCR plates assembling), to avoid operator variability; (4) by using high quality certified qPCR reagents and (5) commercial RG probes (Taqman Gene Expression Assay) properly designed to guarantee maximum PCR efficiency and specificity [37] to allow trustworthy comparison in gene expression stability evaluation. Furthermore, in view of the unsuitability of conventional RGs, such as GAPDH or ACTB, for most of the experimental situations [10,17-20], we chose a microarray data meta-analysis to identify new potential RGs. Since 2 out of 4 of the best performing RGs (ESD and YAP1) identified in the present study belong to the meta-analysis group, we can confirm the reliability of this strategy in the identification of new candidate RGs within a specific experimental condition. A further advantage is the easier identification of candidate RGs expressed at a comparable order of magnitude to target genes. This might result in an improvement of data normalization since, as highlighted by some researchers [38,39], at the same Ct level, reference and target experience the same condition and real-time RT-PCR kinetics with respect to polymerase activation, reaction inactivation, stochastic relation between target and primer concentration, and reaction end product inhibition by the generated RT-PCR product. However, the filtering procedures used to search microarray data for stably expressed genes are biased for low expressed genes.

RG expression stability within sample populations and under experimental condition (tumor versus normal lung specimens) has been evaluated by: (1) descriptive statistic; (2) equivalence test; (3) GeNorm applet. All approaches matched and show that for gene expression profiling in NSCLC the most suitable reference genes to be used for normalization are rRNA18S, POLR2A, ESD and YAP1. The effectiveness of POLR2A, the main enzyme in mRNA transcription, is further supported by others [40]. It is characterized by stable expression among different tissues and it is not modulated by treatment with TPA and ionomycin, indicating resistance to cellular activation. The efficacy of rRNA18S, however, has been criticized because: (1) it is highly expressed, representing up to 80% of cellular RNA and (2) it is transcribed by a specific RNA-polymerase [41]. Nevertheless the results of the present study clearly show that there are no significant differences between rRNA18s and the other best performing RGs identified. In addition, rRNA18s has already been recommended for gene expression analysis in non-microdissected kidney biopsies [10]. A recent assessment of RGs for lung cancer studies by Liu et al. [42] has indicated that GAPDH is the most suitable RG for qPCR studies in NSCLC tissue samples. In our study, however, all the statistical approaches used suggested that GAPDH is far from being a valid RG due to its evident modulation by the neoplastic transformation. Our data are strongly in agreement with previous indication that GAPDH is regulated in response to various stimuli (i.e hypoxia, insulin, mitogen, EGF) and that its transcript is elevated in various cancer tissues [43-45], including lung cancer [46]. The discrepancy with Liu's data could be related to the different ethnical origin of the specimens. Even so, it probably reflects the employment of a different data analysis approach. Liu et al., in fact, defined GAPDH as the optimal RG because its expression was almost equal to the mean expression of the other six endogenous control genes they analyzed. This is not a satisfactory parameter of RG stability because it is closely dependent on the variability of the remaining RGs and could not be used to assess their constancy within investigated samples. Furthermore all their results refer to pooled tumor and normal lung tissues samples and do not consider whether and how high RG expression levels were affected by malignant transformation. The latter assessment is mandatory to warrant the validity of quantification data [47].

## Conclusion

Careful validation of RGs has been repeatedly advocated as mandatory to ensure the accuracy of normalized data in qPCR studies adopting relative quantification. Our results invite the conclusion that for gene expression profiling in NSCLC the RGs most suitable for normalization are rRNA18S, POLR2A, ESD and YAP1. For accurate normalization we recommend their concurrent use since this results in more reliable quantification. Furthermore our results pinpoint the limits of conventional RGs and suggest that relatively simple meta-data analysis of genome wide transcription profiling studies is a useful way of identifying putative RGs.

## Competing interests

The author(s) declare that they have no competing interests.

## Authors' contributions

SS designed the experiments, carried out the qPCR analysis and drafted the manuscript. LIM extracted and quantified RNA samples. CAR performed the meta-analysis of microarray data. CF performed the statistical analysis. NS collected samples and performed clinical characterization, GVS reviewed the manuscript and has given final approval of the version for publication. All authors read and approved the final manuscript.

## Pre-publication history

The pre-publication history for this paper can be accessed here:



## Figures and Tables

**Table 1:** Gene expression assays.

**Reference gene**	**Gene symbol**	**Gene name**	**Assay on Demand**
+	BTF3 *	basic transcription factor 3	Hs00265768_m1
+	ESD *	Esterase D/formylglutathione hydrolase	Hs00382661_m1
+	HIST1H2BC *	histone 1, H2bc	Hs00830401_s1
+	RPL30 *	ribosomal protein L30	Hs00265497_m1
+	YAP1 *	Yes-associated protein 1, 65kDa	Hs00371735_m1
+	POLR2A	polymerase (RNA) II polypeptide A, 220kDa	Hs_00172187_m1
+	PPIA	cyclophilin A	Hs_99999904_m1
+	PGK1	phosphoglycerate kinase 1	Hs_99999906_m1
+	rRNA18S	18S ribosomal RNA	Hs99999901_s1
+	GAPDH	glyceraldehyde-3-phosphate dehydrogenase	Hs_99999905_m1
+	ACTB	β-actin	Hs_99999903_m1
+	RPLP0	ribosomal protein, large, P0	Hs99999902_m1
-	BRCA1	breast cancer 1, early onset	Hs00173237_m1
-	ERCC2	excision repair cross-complementing rodent repair deficiency, complementation group 2	Hs00361161_m1
-	RRM2	ribonucleotide reductase M2 polypeptide	Hs00357247_g1

*indicates RGs selected from microarray data meta-analysis.
